# Detection of extracardiac abnormalities by early comprehensive abdominal ultrasound screening in neonates with congenital heart disease

**DOI:** 10.1007/s00431-026-06772-2

**Published:** 2026-02-02

**Authors:** Takashi Matsumoto, Takahiro Kido, Yuki Okada, Takashi Murakami, Hidetoshi Takada

**Affiliations:** 1 Department of Pediatrics, University of Tsukuba Hospital, Tsukuba, Ibaraki Japan; 2 Department of Child Health, Institute of Medicine, University of Tsukuba, 1-1-1 Tennodai, Tsukuba, Ibaraki 305-8575 Japan

**Keywords:** Abdominal ultrasound screening, Congenital heart disease, Extracardiac abnormality, Malformation syndrome, Neonatal intensive care unit, Perioperative management

## Abstract

Congenital heart disease (CHD) is frequently associated with extracardiac abnormalities that can significantly affect prognosis and management. Although abdominal ultrasound is a readily available and noninvasive tool, detailed observational studies on comprehensive screening for neonates with CHD remain lacking. We aimed to evaluate the prevalence of significant abdominal abnormalities detected during early comprehensive ultrasound screening in neonates with CHD. This single-center, retrospective case series included neonates admitted to a tertiary neonatal intensive care unit (NICU) between April 2021 and May 2024 who underwent screening within 14 days of birth. We compared the proportion of neonates with significant abnormalities and the number of abnormalities per patient between those with and without chromosomal abnormalities or malformation syndromes. Among 59 neonates, significant abnormalities were identified in 29 (49.2%). The most common were hydronephrosis (13.6%), hepatomegaly (10.2%), and intestinal malrotation (8.5%). The proportion of significant abnormalities did not differ significantly between the groups with and without chromosomal abnormalities or malformation syndromes (66.7% vs. 39.5%, *P* = 0.06). However, the number of abnormalities per patient was significantly higher in the former group (1.38 vs. 0.61, *P* = 0.03).

*Conclusion*: Early comprehensive abdominal ultrasound screening in hospitalized neonates with CHD revealed significant abnormalities in 49.2% of cases. This screening may improve systemic management of patients with CHD, including perioperative care, and facilitate individualized treatment of CHD‒with or without chromosomal or malformation syndromes‒by enhancing phenotype characterization.

**Table:** 

**What is Known:** ・*Congenital heart disease (CHD) is frequently associated with extracardiac abnormalities that can affect prognosis and management.* ・*Detailed observational studies on comprehensive abdominal ultrasound screening for neonates with CHD remain lacking.*
**What is New:** ・*Early comprehensive abdominal ultrasound screening in hospitalized neonates with CHD revealed significant abnormalities in 49.2%.* ・*While abnormalities were more frequent in neonates with chromosomal or malformation syndromes, 39.5% of non-syndromic neonates had significant abnormalities; the screening improves systemic management and individualized treatment.*

**Supplementary Information:**

The online version contains supplementary material available at 10.1007/s00431-026-06772-2.

## Introduction

Neonates with congenital heart disease (CHD) often require prolonged stays in the neonatal intensive care unit (NICU) or surgical intervention. Comprehensive systemic and cardiovascular management is essential in these cases. Notably, 20‒28% of children with CHD have extracardiac abnormalities [1–3]. Early detection of these anomalies is critical because their presence significantly influences overall management and the formulation of optimal treatment strategies [4]. Compared with computed tomography (CT) or contrast studies, ultrasound is a readily available, noninvasive, and highly useful tool for neonatal screening. As it can be performed at the bedside [5, 6], ultrasound eliminates the need for patient transport, reduces procedural burden, and minimizes the risk of vital sign instability‒an especially important advantage for neonates with CHD. However, routine comprehensive abdominal ultrasound screening for all neonates with CHD has not yet become standard practice, and to our knowledge, no detailed observational studies have been conducted on this topic to date.

Since 2021, we have performed comprehensive abdominal ultrasound screening for all neonates with CHD admitted to the University of Tsukuba Hospital NICU during the early neonatal period. By accumulating the findings and evaluating their clinical impact, we aimed to generate evidence supporting the role of comprehensive abdominal ultrasound in the management of CHD. The objective of this study was to determine the prevalence of significant abnormalities detected through this screening.

## Methods

### Study design and setting

This single-center retrospective case series was conducted at Tsukuba University Hospital, a level III perinatal center in Japan. Fetuses with CHD from the southern region of Ibaraki Prefecture who may require surgery after birth are typically managed at our hospital, where they undergo fetal echocardiography, planned delivery, and postnatal intensive care.

### Participants

Eligible neonates were those admitted to our NICU between April 1, 2021, and May 31, 2024, diagnosed with CHD, and who underwent comprehensive abdominal ultrasound screening within 14 days of birth. Neonates who underwent screening only after the onset of abdominal symptoms were excluded. As this was a retrospective descriptive study, no formal a priori sample size calculation was performed.

### Comprehensive abdominal ultrasound screening

All screenings were performed by one of two neonatologists (T. Matsumoto or Y. Okada) trained in pediatric abdominal ultrasonography. Screening was performed as early as possible after NICU admission and CHD diagnosis‒generally after completing initial stabilization and routine procedures. Screening was postponed in cases of unstable respiratory or circulatory status due to disease or immaturity, or when ultrasound was deemed clinically inadvisable.

This screening assessed all abdominal organs, including solid organs (liver, gallbladder, common bile duct, pancreas, spleen, and adrenal glands), renal and urinary systems (kidneys, ureters, and bladder), pelvic reproductive organs (uterus and ovaries), gastrointestinal tract (esophagus, stomach, duodenum, small intestine, and colon), and blood vessels (portal vein, superior mesenteric artery, and vein). The testes were evaluated only if cryptorchidism was suspected during physical examination. Intestinal malrotation, commonly associated with CHD [7], was assessed based on three criteria: an abnormal position of the superior mesenteric artery and vein, failure of the horizontal part of the duodenum to cross the midline between the superior mesenteric artery and abdominal aorta, and malpositioning of the ileocecal region outside the right lower quadrant [8, 9]. All examinations were performed using an Aplio i800 ultrasound imaging system (Canon Medical Systems, Tochigi, Japan).

### Data collection

Patient data extracted from electronic medical records included sex, gestational age, birth weight, age at examination, ultrasound findings, primary diagnosis of CHD, diagnosis of chromosomal abnormalities, presence or absence of malformation syndrome, prenatally diagnosed abdominal organ abnormalities, and relevant medical or treatment history. The primary CHD diagnosis was confirmed by a pediatric cardiologist (T. Murakami).

### Outcome measurements

We calculated the prevalence of significant abnormalities and the number of abnormalities per patient. These outcomes were analyzed for the entire study group and separately for patients with and without chromosomal abnormalities or malformation syndromes. Normal subtypes and transient findings in the clinical course (e.g., edematous thickening of the gallbladder wall and biliary sludge) were also considered abnormal findings. Two pediatricians (T. Matsumoto and T. Kido) checked each finding against the patient's clinical information and excluded those that had no influence on the clinical course or were judged as “not significant.” The remaining significant abnormalities were included in the analyses. Analyses used a complete-case approach; no imputation was performed.

### Statistical analyses

We compared the proportion of significant abnormalities between groups using Fisher's exact test and the number of abnormalities per patient using the Mann–Whitney U test. Statistical significance was defined as *P* < 0.05. All analyses were conducted using EZR software [10].

### Ethics

This study was approved by the Ethics Committee of the University of Tsukuba Hospital (R06-122). Study information was disclosed on the hospital website, and an opt-out method was used to allow participants or guardians to decline inclusion. The ethics committee determined that individual informed consent was not required.

## Results

**Fig.1:**
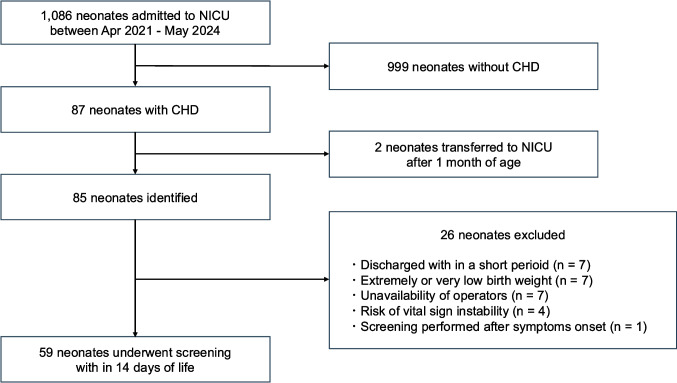
Flowchart detailing the process of identifying and including the study sample

During the study period, 1,086 neonates were admitted to our NICU, of whom 87 (8.0%) had CHD [Fig. 1]. Two were transferred from other hospitals after 1 month of age. Among the remaining 85 neonates, 60 (70.6%) underwent comprehensive abdominal ultrasound screening within 14 days of birth. Of these 60 neonates, one was excluded because the screening was performed after the onset of abdominal symptoms. The final analysis therefore included 59 neonates. Reasons for not performing screening included early discharge (*n* = 7), extremely low or very low birth weight with risk of intraventricular hemorrhage (*n* = 7), unavailability of qualified ultrasound operators (*n* = 7), and risk of vital sign instability (*n* = 4). Characteristics of unscreened neonates are shown in Supplementary Table 1. Among these unscreened neonates, ventricular septal defect (VSD) (*n* = 14) was the most common primary CHD diagnosis.

The median gestational age at birth of the 59 screened neonates was 38 weeks, and the median birth weight was 2,855 g (Table 1). A prenatal diagnosis of CHD was made in 38 neonates (64.4%), and 36 (61.0%) underwent surgery for CHD during their NICU stay. Thirty (50.8%) neonates were screened at birth. Twenty-one (35.6%) had chromosomal abnormalities or malformation syndromes. The most common primary CHD diagnosis was tetralogy of Fallot (23.7%), followed by VSD (16.9%), and coarctation complex (11.9%) (Table 2).

**Table 1:** Characteristics of neonates who underwent early comprehensive abdominal ultrasound screening

	*n* = 59
Gestational age, weeks	38 (28–41)
Birth weight, g	2855 (789–4025)
Sex, male	31 (52.5)
Prenatal diagnosis of CHD	38 (64.4)
Surgery for CHD during NICU stay	36 (61.0)
Age at time of screening, days	0 (0–12)
Genetic syndrome identified + suspected	21 (35.6)
Chromosomal abnormality	17 (28.8)
Trisomy 21	9 (15.2)
Trisomy 18	3 (5.1)
Trisomy 13	2 (3.4)
22q11.2 deletion syndrome	2 (3.4)
1p36 deletion syndrome	1 (1.7)
Malformation syndrome	4 (6.8)

Data are presented as No. (%) or median (min–max). CHD, Congenital heart disease. NICU, Neonatal intensive care unit

**Table 2:** Primary congenital heart disease diagnoses in the study population

Primary CHD diagnosis	*n* (%)
Tetralogy of Fallot	14 (23.7)
Ventricular septal defect	10 (16.9)
Coarctation complex	7 (11.9)
Double outlet right ventricle	4 (6.8)
Pulmonary atresia with intact ventricular septum	4 (6.8)
Borderline left ventricle	3 (5.1)
Transposition of the great arteries	3 (5.1)
Atrioventricular septal defect	2 (3.4)
Common arterial trunk	2 (3.4)
Single ventricle	2 (3.4)
Aortic stenosis	1 (1.7)
Atrial septal defect	1 (1.7)
Coarctation of the aorta	1 (1.7)
Hypoplastic left heart syndrome	1 (1.7)
Patent ductus arteriosus	1 (1.7)
Total anomalous pulmonary venous connection	1 (1.7)
Tricuspid atresia	1 (1.7)
Valvar pulmonary stenosis	1 (1.7)
Total	59

**Fig.2:**
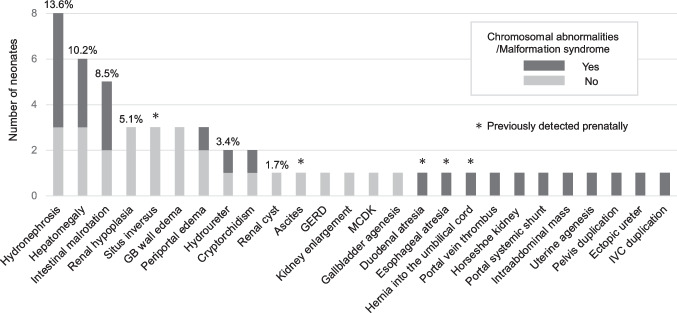
Number and prevalence of significant abnormalities of early comprehensive abdominal ultrasound screening. Cases may have multiple findings. GB, Gall bladder. GERD, Gastroesophageal reflux. MCDK, Multicystic dysplastic kidney. IVC, Inferior vena cava

Comprehensive abdominal ultrasound screening revealed significant abnormalities in 29 of 59 neonates (49.2% [95% CI 35.9%−62.5%]). A detailed list of these findings is provided in Supplementary Table 2. The number of significant abdominal abnormalities was 0 in 30 neonates (50.8%), 1 in 17 (28.8%), 2 in 5 (8.5%), and ≥ 3 in 7 (11.9%) (Table 3). Abnormalities were detected in almost all abdominal organs. The most frequent significant abnormality was hydronephrosis (13.6%), followed by hepatomegaly (10.2%) and intestinal malrotation (8.5%) (Fig. 2). When restricted to congenital malformations alone, abnormalities were identified in 21 of 59 neonates (35.6%). Significant abnormalities identified during the fetal period included situs inversus, ascites, duodenal atresia, esophageal atresia, and umbilical cord hernia. Biliary sludge, intrahepatic bile duct dilation, abnormal hepatic artery course, umbilical cord cysts, and abnormal portal vein course were judged to be not significant [Supp Table 2]. The proportion of significant abnormalities did not differ significantly between the groups with and without chromosomal abnormalities or malformation syndromes (66.7% vs. 39.5%, *P* = 0.06). However, the number of abnormalities per patient was significantly higher in the group with chromosomal abnormalities or malformation syndromes (1.38 vs. 0.61, *P* = 0.03) (Table 3). Six neonates had isolated VSD without chromosomal abnormalities or malformation syndromes, and none had any abnormalities.

**Table 3:**
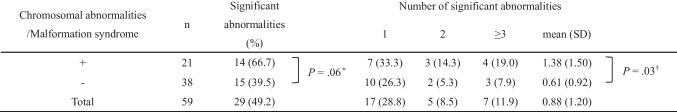
Comparison of significant abnormalities in the presence or absence of chromosomal abnormalities or malformation syndromes

Data are presented as No. (%). ＊*P* value is based on Fisher's exact test. †*P* value is based on Mann-Whitney U Test

## Discussion

In this study, 49.2% of the 59 neonates were found to have significant abnormalities on comprehensive abdominal ultrasound screening within 14 days of birth. Neonates with chromosomal abnormalities or malformation syndromes tended to have more than two significant abdominal abnormalities. To our knowledge, this is the first study to report the prevalence of each significant abnormality detected by abdominal ultrasound screening in neonates with CHD admitted to the NICU. Few previous studies have performed such a comprehensive screening in this population. Gonzalez et al. conducted abdominal ultrasound screening of 131 neonates with CHD admitted to the NICU; however, they did not report the prevalence of each specific significant abnormality [11]. The present study thus has important implications for promoting the use of comprehensive abdominal ultrasound screening in neonates with CHD.

The proportion of significant abnormalities in our study was higher than those reported previously. Rosa et al. performed abdominal ultrasound screening in 164 children with CHD admitted to the PICU (mean age, 8.4 months) and found significant abnormalities in 12.2%, including renal and urinary tract malformations and situs inversus [12]. Gonzalez et al. reported a prevalence of 36.6% [11]. The higher proportion in our study may be attributed to two main factors. First, the proportion of mild CHD, such as atrial septal defect (ASD), ventricular septal defect (VSD), and patent ductus arteriosus (PDA), was relatively low (20.3%). Among the 10 neonates with VSD, significant abnormalities were identified in only two cases. Although the sample size was limited, these findings suggest that mild CHD may be associated with a lower prevalence of abdominal abnormalities. Second, the use of high-performance ultrasound equipment may have increased the detection rate. Compared with previous studies, ours identified a broader range of significant abdominal abnormalities, likely reflecting the superior sensitivity of the ultrasonography system employed.

Early comprehensive abdominal ultrasound screening in neonates with CHD offers several benefits, particularly in systemic and perioperative management. In our study, significant abnormalities were detected in 39.5% of neonates without chromosomal abnormalities or malformation syndromes. Notably, hydronephrosis‒associated with a risk of urinary tract infection‒was detected in 16.9% of neonates, aiding in the management of febrile episodes. Additionally, intestinal malrotation was identified in 8.5% of patients, enabling early recognition of the risk of midgut volvulus. In one case of tetralogy of Fallot with trisomy 21, intestinal malrotation was detected on screening at birth, and midgut volvulus developed within 1 week. This patient presented with vomiting, and timely emergency surgical intervention could be performed owing to prior detection via screening. Neonates with CHD often require early surgical intervention during the early postnatal period. Extracardiac complications may cause hemodynamic instability and even delay cardiac surgery. Identifying these risks in advance by early comprehensive abdominal ultrasound screening enhances emergency response to symptomatic events and consequently contributes to perioperative systemic management.

Early detection of extracardiac intra-abdominal abnormalities also contributes to individualized CHD management in neonates with chromosomal abnormalities or malformation syndromes. For such patients, treatment should not rely solely on standard CHD protocols. Instead, therapeutic goals should consider the life expectancy and neurological prognosis associated with the underlying syndromes, sometimes limiting cardiac surgery to palliative procedures. Rapid diagnosis of chromosomal abnormalities and malformation syndromes is therefore crucial for planning appropriate strategies. Although genetic methods such as G-banding, FISH, microarray, whole-exome sequencing, and whole-genome analysis are available, careful phenotypic characterization remains essential [13]. Our study showed a high frequency of significant abnormalities across various organs in neonates with CHD, as well as chromosomal abnormalities or malformation syndromes, suggesting that abdominal ultrasound is an optimal modality for multi-organ phenotypic screening.

However, early comprehensive abdominal ultrasound screening may have limited utility in certain subgroups. In this study, abdominal ultrasound findings were normal in all neonates with isolated VSD without chromosomal abnormalities or malformation syndromes. Furthermore, many patients with mild CHD, particularly those suitable for early discharge, typically require only observation and standard follow-up, obviating the need for comprehensive management or individualized treatment. Considering resource allocation, restricting early comprehensive abdominal ultrasound screening to high-risk neonates may represent a more cost-effective approach, particularly in cases of moderate-to-severe CHD requiring surgery or prolonged hospitalization, prenatal extracardiac abnormalities, or suspected chromosomal abnormalities or malformation syndromes.

This study had several limitations. First, it included only inpatients, excluding mild CHD cases managed on an outpatient basis. Additionally, due to the retrospective design, early comprehensive abdominal ultrasound screening was not performed in some eligible neonates. Since mild CHD was more frequent among unscreened neonates, the prevalence of significant abnormalities in this study may have been overestimated. Indeed, the proportion of significant abnormalities was low in screened mild CHD cases. Therefore, our study provides evidence for the clinical significance of comprehensive abdominal ultrasound screening in patients with moderate-to-severe CHD; however, its application to the entire population of neonates with all types of CHD requires further investigation. Second, some significant abnormalities, such as hepatomegaly or gallbladder wall edema, resolved after CHD surgery and may represent physiological changes rather than congenital malformations. Nonetheless, we believe that these findings warrant inclusion in the analysis of the prevalence of significant abnormalities, owing to their clinical significance. Notably, the prevalence of abnormalities remained high at 35.6% even when restricted to congenital malformations alone, further underscoring the clinical importance of this screening. Third, screening was performed by individual neonatologists, raising the possibility of inter-observer variability. This highlights the need for a standardized protocol for comprehensive abdominal ultrasound screening to ensure consistency. Finally, the use of highly experienced operators and high-performance equipment may limit the generalizability of our findings to settings with different resources and expertise levels. Future prospective multicenter studies that include outpatients are warranted to overcome these limitations and validate these results. Furthermore, the impact of early comprehensive abdominal ultrasound screening on the duration of hospitalization for neonates with CHD remains a subject for future research.

In conclusion, early comprehensive abdominal ultrasound screening of hospitalized neonates with CHD identified significant abnormalities in 49.2% of cases. This screening enabled early detection of abnormalities that could affect systemic and perioperative management. It also facilitated phenotypic characterization necessary for genetic evaluation in neonates with chromosomal abnormalities or malformation syndromes, supporting earlier diagnosis of these underlying syndromes, which consequently contributes to the optimization of individualized treatments for CHD.

## Supplementary Information

Below is the link to the electronic supplementary material.

Supplementary file1 (DOCX 40 KB)

Supplementary file2 (DOCX 28 KB)

## Data Availability

Datasets are available from the corresponding author upon reasonable request.

## Abbreviations

CHD: Congenital heart disease
CT: Computed tomography
NICU: Neonatal intensive care unit
VSD: Ventricular septal defect
ASD: Atrial septal defect
PDA: Patent ductus arteriosus
