# Supplementary material for: Detection of extracardiac abnormalities by early comprehensive abdominal ultrasound screening in neonates with congenital heart disease
Source: Eur J Pediatr. 2026 Feb 2;185(2):116. doi: 10.1007/s00431-026-06772-2 (PMC12864229; doi:10.1007/s00431-026-06772-2)
Supplement: Supplementary file 1 — Supplementary file1 (DOCX 40 KB) [file 431_2026_6772_MOESM1_ESM.docx]

| **Supplementary Table 1** Clinical characteristics, primary congenital heart disease diagnoses of neonates  not undergoing early comprehensive abdominal ultrasound screening | | | | | | |
| --- | --- | --- | --- | --- | --- | --- |
| Sex | Gestaional age (weeks) | Birth weight (g) | Chromosomal abnormality | Malformation syndrome | Primary CHD diagnosis | Reasons for not performing screening |
| F | 32 | 800 | Trisomy 18 | - | DORV | Extremely or very low birth weight |
| F | 36 | 1263 | Trisomy 18 | - | HLHS | Extremely or very low birth weight |
| M | 31 | 728 | Trisomy 18 | - | VSD | Extremely or very low birth weight |
| M | 35 | 1103 | 3q deletion syndrome | - | TOF | Extremely or very low birth weight |
| F | 33 | 1484 | - | - | VSD | Extremely or very low birth weight |
| M | 22 | 541 | - | - | VSD | Extremely or very low birth weight |
| F | 31 | 919 | - | - | VSD | Extremely or very low birth weight |
| M | 38 | 2820 | - | - | VSD | Discharge within a short period |
| M | 38 | 2010 | - | - | VSD | Discharge within a short period |
| F | 40 | 3020 | - | - | VSD | Discharge within a short period |
| M | 38 | 2080 | - | - | VSD | Discharge within a short period |
| M | 36 | 2810 | - | - | VSD | Discharge within a short period |
| M | 35 | 2490 | - | - | VSD | Discharge within a short period |
| M | 39 | 3348 | - | - | VSD | Discharge within a short period |
| M | 38 | 3059 | - | - | DOMV | Unavailability of operators |
| F | 38 | 2965 | - | - | DORV | Unavailability of operators |
| M | 35 | 1703 | - | - | VSD | Unavailability of operators |
| M | 37 | 2254 | - | - | TOF | Unavailability of operators |
| F | 39 | 2885 | - | - | TOF | Unavailability of operators |
| M | 36 | 1851 | - | - | VSD | Unavailability of operators |
| M | 33 | 1511 | - | - | VSD | Unavailability of operators |
| M | 39 | 2941 | - | - | HLHS | Risk of vital sign instability |
| M | 38 | 3005 | - | - | AVSD | Risk of vital sign instability |
| F | 37 | 2456 | - | - | TAPVC | Risk of vital sign instability |
| M | 38 | 2757 | - | - | SV | Risk of vital sign instability |
| M | 38 | 3505 | - | - | DORV | Screening performed after symptoms onset |

AVSD, Atrioventricular septal defect; borderline LV, Borderline left ventricle; DORV, Double outlet right ventricle; DOMV, Double orifice mitral valve; HLHS, Hypoplastic left heart syndrome; SV, Single ventricle; TAPVC, Total anomalous pulmonary venous connection; TOF, Tetralogy of Fallot; VSD, Ventricular septal defect.
