# Supplementary material for: Detection of extracardiac abnormalities by early comprehensive abdominal ultrasound screening in neonates with congenital heart disease
Source: Eur J Pediatr. 2026 Feb 2;185(2):116. doi: 10.1007/s00431-026-06772-2 (PMC12864229; doi:10.1007/s00431-026-06772-2)
Supplement: Supplementary file 2 — Supplementary file2 (DOCX 28 KB) [file 431_2026_6772_MOESM2_ESM.docx]

| **Supplementary Table S2** Clinical characteristics, primary congenital heart disease diagnoses, and abnormalities detected by early comprehensive abdominal ultrasound screening | | | | | | | |
| --- | --- | --- | --- | --- | --- | --- | --- |
| Sex | Gestaional age (weeks) | Birth weight (g) | Chromosomal abnormality | Malformation syndrome | Age at time of screening (days) | Primary CHD diagnosis | Abnormalities detected by comprehensive abdominal ultrasound screening |
| F | 32 | 2000 | - | - | 4 | AS | Renal hypoplasia, Intestinal malrotation, Ascites^＊^ |
| F | 38 | 2346 | - | - | 8 | borderline LV |  |
| M | 40 | 3718 | - | - | 0 | borderline LV |  |
| M | 38 | 2855 | - | - | 0 | CAT | Intrahepatic bile duct dilatation^†^ |
| M | 38 | 2975 | - | - | 10 | CoA |  |
| M | 37 | 2146 | - | - | 1 | CoA complex | Renal hypoplasia |
| M | 38 | 3011 | - | - | 1 | CoA complex | Edematous thickening of the gallbladder wall |
| F | 40 | 2863 | - | - | 0 | CoA complex |  |
| M | 40 | 3312 | - | - | 13 | CoA complex |  |
| M | 38 | 2815 | - | - | 3 | CoA complex |  |
| F | 41 | 4025 | - | - | 0 | DORV | Intestinal malrotation, Situs inversus^＊^ |
| M | 38 | 3483 | - | - | 0 | DORV |  |
| F | 38 | 3347 | - | - | 1 | PA-IVS | Hepatomegaly |
| M | 38 | 2178 | - | - | 0 | PA-IVS | Hepatomegaly, Periportal edema, Edematous thickening of the gallbladder wall |
| M | 38 | 3255 | - | - | 6 | PA-IVS | Hepatomegaly |
| M | 37 | 2878 | - | - | 1 | SV | Situs inversus^＊^ |
| M | 37 | 3122 | - | - | 7 | SV |  |
| M | 38 | 2902 | - | - | 3 | TAPVC | Periportal edema, Kidney enlargement, Edematous thickening of the gallbladder wall |
| F | 40 | 2683 | - | - | 0 | TGA |  |
| M | 37 | 2602 | - | - | 1 | TGA |  |
| F | 38 | 3007 | - | - | 1 | TGA |  |
| M | 39 | 3300 | - | - | 0 | TOF | Situs inversus^＊^ |
| F | 35 | 1961 | - | - | 0 | TOF |  |
| F | 37 | 3340 | - | - | 0 | TOF | Hydronephrosis, MCDK |
| F | 40 | 2870 | - | - | 1 | TOF |  |
| M | 37 | 2983 | - | - | 1 | TOF | Hydronephrosis |
| M | 39 | 3188 | - | - | 2 | TOF | Gastroesophageal reflux |
| F | 40 | 2736 | - | - | 0 | TOF |  |
| F | 38 | 2024 | - | - | 4 | TOF |  |
| F | 39 | 2883 | - | - | 3 | TOF | Gallbladder agenesis |
| M | 38 | 2977 | - | - | 1 | TrA | Hydronephrosis |
| M | 39 | 3190 | - | - | 0 | vPS |  |
| F | 38 | 3208 | - | - | 0 | VSD |  |
| M | 33 | 1176 | - | - | 4 | VSD |  |
| F | 34 | 2092 | - | - | 0 | VSD |  |
| F | 38 | 2905 | - | - | 0 | VSD |  |
| F | 40 | 2845 | - | - | 0 | VSD |  |
| F | 37 | 2369 | - | - | 0 | VSD |  |
| F | 37 | 1341 | 1p36 deletion syndrome | - | 1 | CoA complex | Hydronephrosis, Uterine agenesis, Portal vein malformation^†^ |
| M | 37 | 2801 | 22q11.2 deletion syndrome | - | 7 | PA-IVS | Hydronephrosis, Cryptorchidism, Hepatic artery malformation^†^ |
| F | 38 | 2725 | 22q11.2 deletion syndrome | - | 0 | TOF | Hepatomegaly |
| F | 38 | 1781 | Trisomy 13 | - | 0 | DORV | Intraabdominal mass |
| F | 39 | 2870 | Trisomy 13 | - | 0 | VSD | Horseshoe kidney, Pelvis duplication, Hydroureter, Ectopic ureter, Inferior vena cava duplication |
| M | 32 | 904 | Trisomy 18 | - | 0 | AVSD | Hepatomegaly |
| M | 28 | 789 | Trisomy 18 | - | 7 | CoA complex |  |
| M | 37 | 1669 | Trisomy 18 | - | 0 | DORV | Hydronephrosis, Esophageal atresia^＊^ |
| M | 37 | 3445 | Trisomy 21 | - | 0 | ASD | Intestinal malrotation, Umbilical cord cysts^†^ |
| F | 40 | 2734 | Trisomy 21 | - | 0 | AVSD | Biliary sludge^†^ |
| M | 39 | 2518 | Trisomy 21 | - | 0 | TOF |  |
| M | 40 | 3518 | Trisomy 21 | - | 0 | TOF | Hepatomegaly, Periportal edema, Intestinal malrotation |
| F | 38 | 2600 | Trisomy 21 | - | 5 | TOF | Hydronephrosis |
| F | 37 | 2352 | Trisomy 21 | - | 1 | TOF |  |
| M | 38 | 3262 | Trisomy 21 | - | 0 | VSD |  |
| M | 37 | 2328 | Trisomy 21 | - | 0 | VSD | Hernia into the umbilical cord^＊^ |
| M | 37 | 2470 | Trisomy 21 | - | 0 | VSD |  |
| F | 38 | 3887 | - | + | 1 | borderline LV | Portal vein thrombus, Hydronephrosis, Intestinal malrotation, Portal systemic shunt |
| F | 35 | 1986 | - | + | 0 | CAT | Duodenal atresia^＊^ |
| F | 38 | 2782 | - | + | 1 | HLHS |  |
| M | 38 | 2902 | - | + | 12 | PDA | Renal cyst, Hydroureter, Renal hypoplasia, Cryptorchidism |

AS, Aortic stenosis; ASD, Atrial septal defect; AVSD, Atrioventricular septal defect; borderline LV, Borderline left ventricle; CAT, Common arterial trunk;

CoA, Coarctation of the aorta; CoA complex, Coarctation complex; DORV, Double outlet right ventricle; HLHS, Hypoplastic left heart syndrome; PA‑IVS, Pulmonary atresia with intact ventricular septum; PDA, Patent ductus arteriosus; SV, Single ventricle; TAPVC, Total anomalous pulmonary venous connection; TGA, Transposition of the great arteries; TOF, Tetralogy of Fallot; TrA, Tricuspid atresia; vPS, Valvar pulmonary stenosis; VSD, Ventricular septal defect.

^＊^Previously detected prenatally.

^†^Non-pathological abnormalities.
